# Cannabigerol (CBG) Modulates Neutrophil Activity and Ameliorates Rheumatoid Arthritis Pathogenesis

**DOI:** 10.3390/ph19040560

**Published:** 2026-03-31

**Authors:** Miran Aswad, Antonina Pechkovsky, Haya Hamza, Igal Louria-Hayon

**Affiliations:** 1 The Shanti Center for Medical Cannabis Research, Rambam Health Care Campus, Haifa 3109601, Israel; 2 Clinical Research Institute at Rambam (CRIR), Rambam Health Care Campus, Haifa 3109601, Israel; 3 The Division of Research at Rambam, Rambam Health Care Campus, Haifa 3109601, Israel

**Keywords:** rheumatoid arthritis, neutrophils, cannabigerol (CBG), cannabis, cannabinoids

## Abstract

**Background/Objectives**: Rheumatoid arthritis (RA) is a chronic, inflammatory, autoimmune disease that primarily affects the joints. Current treatments aim to relieve pain and limit joint damage; however, many are associated with significant side effects or high costs. Neutrophils play a critical role in RA development and progression by driving synovial inflammation and tissue damage, yet no approved therapies directly target neutrophil-mediated pathogenic mechanisms. Cannabinoids have demonstrated anti-inflammatory potential. Although cannabinoids have been studied in RA, the direct modulation of neutrophil-driven mechanisms by purified CBG has not been systematically addressed. To harness the cannabinoid potential, we investigated the effects of the purified cannabinoid Cannabigerol (CBG) on neutrophil-mediated immune responses in RA. **Methods**: We assessed the effects of CBG on human blood isolated neutrophil cytokine secretion, signal transduction and migration as ex vivo models. In addition, collagen antibody-induced arthritis (CAIA) was applied in C57BL/6 wt mice, and immune-cell recruitment and cytokine secretion were examined after CBG treatment. **Results**: Ex vivo experiments demonstrated that CBG hampered the secretion of pro-inflammatory cytokines from human neutrophils in a dose-dependent manner (TNF-α and IL-6 by 68% and 72%, respectively). Furthermore, CBG downregulated inflammatory signal transduction, such as P38-MAPK, ERK1/2 and Akt phosphorylationpost neutrophil activation by 41%, 54% and 78%, respectively. Importantly, 60% of the CBG downregulation of IL-6 was consistent with the CB2 receptor axis in a selective way. In addition, CBG attenuated neutrophil migration toward IL-8 by 67%. To further evaluate CBG therapeutic capacity, we used CAIA as an in vivo model. CBG treatment resulted in improving mice arthritis clinical scores and body weight in comparison to RA-diseased mice. Moreover, CBG reduced leukocyte recruitment to the inflamed joints by 48%, primarily through the inhibition of neutrophil and monocyte cells to 27% and 49%, respectively. Additionally, CBG showed its anti-inflammatory effect by decreasing inflammatory cytokines like IL-6 and IL-1β by 98% and 60% in the blood. Also, CBG reduced MCP-1 and IL-1β cytokines in the joints by 22% and 38%, respectively. **Conclusions:** These results show that CBG has anti-inflammatory capacity and therapeutic potential in regulating neutrophil-mediated immunity in RA. These findings are preclinical and require further validation before therapeutic positioning.

## 1. Introduction

Rheumatoid arthritis (RA) is a chronic, inflammatory, autoimmune disease that primarily affects the joints and is associated with autoantibodies that target various molecules including modified self-epitopes [1]. The innate immune system, including toll-like receptors (TLRs), triggers the initial inflammatory response in joint tissues. The synovium, normally a thin membrane lining the joint, becomes inflamed and thickened due to the influx of immune cells. Neutrophils, monocytes/macrophages, mast cells, T and B lymphocytes and other immune cells infiltrate the synovium. The inflamed synovium experiences new blood vessel growth (angiogenesis). Monocytes and macrophages differentiate into osteoclasts, which break down bones. These cells release cytokines and other inflammatory molecules, further amplifying the inflammatory response. The inflammation leads to cartilage and bone destruction [2].

Growing evidence indicates that synovial pathology and systemic inflammation in chronic immune-mediated diseases are sustained by convergent innate–immune and stress-response circuits, making them attractive pharmacological entry points. For example, synovial cell survival programs can be tuned through the ADORA2B–PI3K/Akt/mTOR axis, which has been linked to reduced apoptosis/autophagy in inflammatory joint settings [3]. Macrophage polarization and inflammasome control also emerge as central levers, as Substance P–driven M2 skewing can attenuate tissue inflammation via NF-κB/NLRP3 regulation [4], and NLRP3 activity can be restrained by ubiquitin–proteostasis mechanisms that limit pyroptosis and downstream inflammatory amplification [5]. In RA specifically, blocking the IL-23/IL-17/NF-κB axis has been shown to alleviate synovial inflammation, underscoring the importance of cytokine network interception in disease modification [6]. Beyond canonical cytokine signaling, mechanosensitive channels such as Piezo1 can shape fibroblast inflammatory phenotypes, supporting the concept that biophysical cues integrate with immune pathways in fibrotic/inflammatory microenvironments [7]. Systems-level approaches further reinforce multi-target immunoregulation, including network pharmacology coupled with in vivo validation for complex formulations that mitigate chronic inflammatory remodeling [8], as well as macrophage-targeted nanomedicine platforms designed to reprogram innate immunity for the durable control of inflammation [9]. Mechanistic parallels across immune diseases strengthen translational relevance: TLR4–NF-κB activation driven by upstream regulators has been implicated in inflammatory bowel disease, highlighting conserved innate–inflammatory wiring that may be therapeutically repurposed [10]. Likewise, mitochondrial stress and endothelial dysfunction are increasingly recognized as inflammatory amplifiers that can be ameliorated by metabolic resilience pathways such as SIRT3 signaling [11]. Finally, the growing appreciation that immune dysregulation intersects with neurodegenerative pathobiology emphasizes the broad value of immunomodulatory small molecules that can recalibrate inflammatory set points across tissues [12]. Collectively, these advances provide a strong conceptual basis for testing new candidates to modulate key innate effector functions—particularly neutrophil signaling, cytokine output and trafficking—to interrupt RA-relevant inflammatory cascades.

Of all cells implicated in the pathology of RA, neutrophils possess the greatest cytotoxic potential, owing to their ability to release degradative enzyme, reactive oxygen species and inflammatory cytokines [13]. Also, neutrophil extracellular traps (NETs) are a source of citrullinated autoantigens in RA [14]. NET components act as danger-associated molecular patterns (DAMPs) to activate NLRP3 inflammasomes and the complements in effector lymphocytes, amplifying inflammation. NETs promote the RA-related autoantibody production in B cells, such as anti-citrullinated protein antibodies (ACPAs) and rheumatoid factor (RF), fueling autoimmunity, while ACPAs further induce NETosis, creating a vicious feedback loop. NETs facilitate the release of pro-inflammatory cytokines (e.g., IL-6, IL-1β, TNF-α), exacerbating joint damage. Finally, NETs activate T cells, dendritic cells, and macrophages via boosting the RAGE/TLR9 pathway, thereby driving the proliferation and migration of fibroblast-like synoviocytes [15]. Furthermore, the inflammatory sites are characterized by low levels of oxygen and glucose and high levels of reductive metabolites. Hypoxia causes an inhibition of neutrophil apoptosis in human and murine neutrophils. Neutrophils possess the hypoxia-inducible factor (HIF)-1α and factor inhibiting HIF (FIH) hydroxylase oxygen-sensing pathway that contribute to synovial neutrophil survival in hypoxia despite systemic treatment [16]. Therefore, neutrophils play roles as both primary drivers and chronic amplifiers that make them an attractive target in rheumatoid arthritis, as they play a central role in driving joint inflammation.

Treatment aims to reduce pain and prevent or slow further joint damage. Early treatment with disease-modifying anti-rheumatic drugs (DMARDs) is crucial for achieving remission and preventing irreversible damage. Key treatment approaches include DMARDs, often combined with non-steroidal anti-inflammatory drugs (NSAIDs) and/or corticosteroids, and, in some cases, biological treatments like TNF-α, IL-1 and IL-6 blockers [17]. A new study shows Trimetazidine (TMZ) as an antirheumatic candidate, offering anti-inflammatory effects and a synergistic effect with Methotrexate (MTX) that together reduce the adverse effects of MTX while improving therapeutic efficacy [18].

To date, there is no treatment that specifically targets neutrophils, but the current standard therapeutic approaches show an indirect beneficial effect on their pathogenic profile. RA current treatments prevent neutrophil chemotaxis and migration and decrease degranulation and ROS production. In addition, treatments attenuate the production of inflammatory mediators and prevent the release of NETs [19].

Cannabinoids produce more than 100 naturally occurring chemicals, the most abundant of which are Δ-9-tetrahydrocannabinol (THC), cannabidiol (CBD), terpenes and flavonoids. THC and CBD bind with cannabinoid receptors (CB1 and CB2), which are present in the brain, immune system and many organs [20]. THC and CBD help to manage conditions ranging from chronic pain persistent inflammation, cancer, inflammatory bowel disease, and neurological disorders to even viral diseases such as Human Immunodeficiency virus (HIV) and SARS-CoV-2 [21]. Both THC and CBD exhibit promising therapeutic properties; however, impairments and the increased incidence of mental health diseases are associated with acute and chronic THC use, respectively, and significant side effects are associated with the chronic use of high-dose CBD [22]. Preclinical in vitro and in vivo studies show promising results regarding the anti-arthritic properties of cannabinoids, psychoactive and non-psychoactive alike [23]. Our previous studies demonstrated that a high-CBD extract (CBD-X) significantly reduced pro-inflammatory cytokine secretion in human-derived PBMCs, neutrophils and T cells [24]. More recent findings further show that CBD-X exerts anti-inflammatory effects in rheumatoid arthritis by inhibiting pro-inflammatory cytokine secretion, limiting immune-cell recruitment and attenuating inflammatory signal transduction [25]. However, CBD-X is an extract that has 37% CBD, 1.7% THC, 0.3% CBG and other unknown terpenes and flavonoids [25]. Therefore, we decided to focus our research on purified cannabinoids.

Cannabigerol (CBG) serves as the precursor molecule for the most abundant cannabinoids. While THC and CBD have been more widely studied than CBG, it has had increased attention due to the wide range of potential health benefits [26]. Studies indicate that CBG may have therapeutic potential in treating neurologic disorders (e.g., Huntington disease, Parkinson disease and multiple sclerosis) and inflammatory bowel disease, as well as having antibacterial activity [27]. Moreover, there are anti-inflammatory effects of CBG in rheumatoid arthritis synovial fibroblasts and peripheral blood mononuclear cell cultures [28]. Furthermore, research showed CBG has potential anti-inflammatory properties, including the diabetic kidney disease progression of rats subjected to a high-fat, high-sucrose diet [29].

CBG is a multi-target player that interacts with the endocannabinoid system and other key signaling pathways, such as CB1, CB2, transient receptor potential (TRP) channels and α2-adrenoceptor, potentially influencing inflammation, pain, neurodegeneration and other ailments. CB1 and CB2 are the two main types of cannabinoid receptors. Unlike THC and CBD, CBG does not bind directly to the CB1 receptors in the brain, which are responsible for the psychoactive effects of cannabis. CBG weakly agonizes the action of CB1 and partially acts as an agonist on CB2 receptor agonists [26]. Cannabinoid receptor 2 deficiency exacerbates inflammation and neutrophil recruitment [30]. The expression of the CB2 mRNA can be detected in neutrophils [31]. The CB2 receptor is primarily expressed only when there is active inflammation. Accordingly, CBG’s interaction with CB2 downregulates the inflammation [32]. Furthermore, CBG interacts with TRP channels, which play a crucial role in regulating the cytoplasmic calcium concentration from the extracellular sources as well as the calcium stored within the endoplasmic reticulum (ER). CBG serves as a strong agonist of TRPA1 and a weaker agonist of TRPV1, TRPV2 and TRPV4 while antagonizing TRPM8 (TRP melastatin type 8) [26]. CBG acts as a potent agonist of Peroxisome proliferator-activated receptor gamma (PPARγ), a transcription factor that suppresses NFκB or MAP kinases [33]. In addition, CBG is a potent serotonin 1A receptor (5-HT1A) antagonist, which is known to reduce the activity of NF-κB and the phosphorylation of its inhibitor α (IκB- α), resulting in a decrease in cytokine secretion [29].

Recent computational modeling indicates that CBG possesses a unique 3D conformational flexibility, characterized by ‘coiled’ and ‘stapled’ poses that are sterically distinct from the rigid bicyclic structure of CBD [34]. In addition, CBG exhibits affinity and activity characteristics between Δ9-THC and CBD at the cannabinoid receptors but appears to be unique in its interactions with the α-2 adrenoceptors, PPARγ and 5-HT1A [27].

However, CBG shows anti-inflammatory effects, but due to the lack of research, it is not widely used. Its physicochemical properties lead to considerable challenges, such as poor intestinal absorption, extensive first-pass metabolism, low bioavailability and variable brain penetration, all of which limit its effective application in clinical settings [35]. In addition, the regulatory status of cannabis, sources for cannabis and funding to support studies are difficult barriers to navigate [36].

There is growing interest in CBG and its promising therapeutic potential in treating immune-related diseases. Our aim in this study is to examine the regulatory effect of CBG in mitigating inflammatory RA disease severity through regulating neutrophil activity. We hypothesized that CBG through CB2 receptor mediation directly suppresses neutrophil activation and recruitment, thereby attenuating RA severity.

## 2. Results

### 2.1. CBG Downregulates TNF-α and IL-6 Secretion by Human-Neutrophils

Neutrophils are one of the first lines of host defense against pathogens, and they are involved in the early recognition and killing of infectious pathogens [37]. Synovial pathology reports reveal lymphocyte-predominant infiltration in most RA cases, with synovial neutrophils (SNs) observed in only 30% of patients. However, elevated neutrophil presence in RA synovium correlates with heightened clinical disease activity and an exacerbated inflammatory state [38]. Activated neutrophils function as a source of cytokines that initiate the inflammatory process, including TNF-α [39] and IL-6 [40]. Both cytokines play crucial roles in the activity and severity of RA [41].

Furthermore, Lipopolysaccharide (LPS) is an exogenous endotoxin; it is the canonical agonist for Toll-like Receptor 4 (TLR4) [42]. TLRs recognize Pathogen Associated Molecular Patterns (PAMPs) expressed on microbial pathogens or Danger Associated Molecular Patterns (DAMPs), which may be expressed by cells under stress [43]. In the context of RA pathology, TLR4 plays a pivotal role by recognizing endogenous DAMPs [44]. By utilizing LPS, we were able to induce a highly synchronized and reproducible activation of the TLR4, which effectively mimics the DAMP-driven sterile inflammation seen during RA.

**Figure 1:**
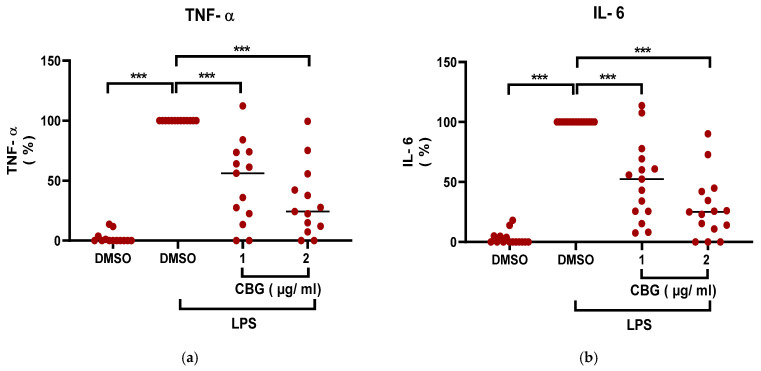
CBG downregulates TNF-α and IL-6 secretion by human Neutrophils. Isolated CBG- treated neutrophils were activated with LPS and TNF-α and IL-6 levels were quantified by ELISA (**a**,**b**). Data represent the mean values obtained from 13 donors for TNF-α and 15 donors for IL-6, normalized to the activated control group (LPS + DMSO). Activated control is considered 100% and all other values were calculated relative to it. Statistical analysis was performed using one-way ANOVA; *** *p* < 0.001 were considered statistically significant.

CBG is a non-psychoactive cannabinoid that exhibits anti-inflammatory activities [26]. To determine whether CBG exerts inhibitory effects on neutrophil-driven inflammation, human peripheral blood neutrophils were isolated (purity ~95%, (Supplementary Figure S1)), treated with CBG and activated with LPS. The secretion of pro-inflammatory cytokine levels of TNF-α and IL-6 was determined. CBG treatment reduced the levels of both cytokines from neutrophils in a significant dose-dependent manner by 52% (48 ± 33.8, *p* < 0.001, *n* = 13, 95% CI (33.86, 69.99)) and 68% (32 ± 29.1, *p* < 0.001, *n* = 15, 95% CI (49.7, 85.8)) of TNF-α levels (Figure 1a) and by 50% (50 ± 32.1, *p* < 0.001, *n* = 13, 95% CI (34.06, 64.9)) and 72% (28 ± 25.3, *p* < 0.001, *n* = 15, 95% CI (56.25, 87.1))of IL-6 levels (Figure 1b). The shown results are relative to the normalized activated control treatment group (LPS, DMSO).

To assess whether the effects of CBG were influenced by reduced cell viability, neutrophil viability was evaluated using the Alamar Blue assay (Supplementary Figure S2). No significant reduction in cell viability was observed at the CBG concentrations applied in the experiments, whereas decreased viability was detected only at higher concentrations. Accordingly, the observed reduction in cytokine secretion is unlikely to be a consequence of cytotoxicity and is instead consistent with a direct modulatory effect of CBG on neutrophil function.

Consequently, CBG reduces the inflammatory output of human neutrophils by suppressing TNF-α and IL-6 production.

### 2.2. CBG Attenuates Pro-Inflammatory Signaling in Human Neutrophils

Both infectious and non-infectious stimuli, as well as cellular damage, activate inflammatory cells and initiate downstream inflammatory signaling pathways [45]. The exposure of neutrophils to LPS results in the phosphorylation and activation of a p38 mitogen-activated protein (MAP) kinase [46]. In addition, ERK1/2 plays an essential role downstream of immune receptors to elicit inflammatory gene expression in response to infection and cell or tissue damage [47]. Akt is predominantly expressed in the innate immune cells, including neutrophils, macrophages and dendritic cells (DCs), which is critical to the inflammatory response and, more specifically, to innate immune cell development and function [48]. In addition, Akt is a master regulator of the regulatory network required for sensing and responding to the chemoattractant gradient that mediates chemotaxis and aggregation [49]. NF-κB signaling is a pivotal pathway in mediating inflammatory responses that is regulated by PI3K/AKT, MAPK and TLR signaling [50]. Candidates for the disruption of these signaling pathways leads to finding potential treatment targets.

**Figure 2:**
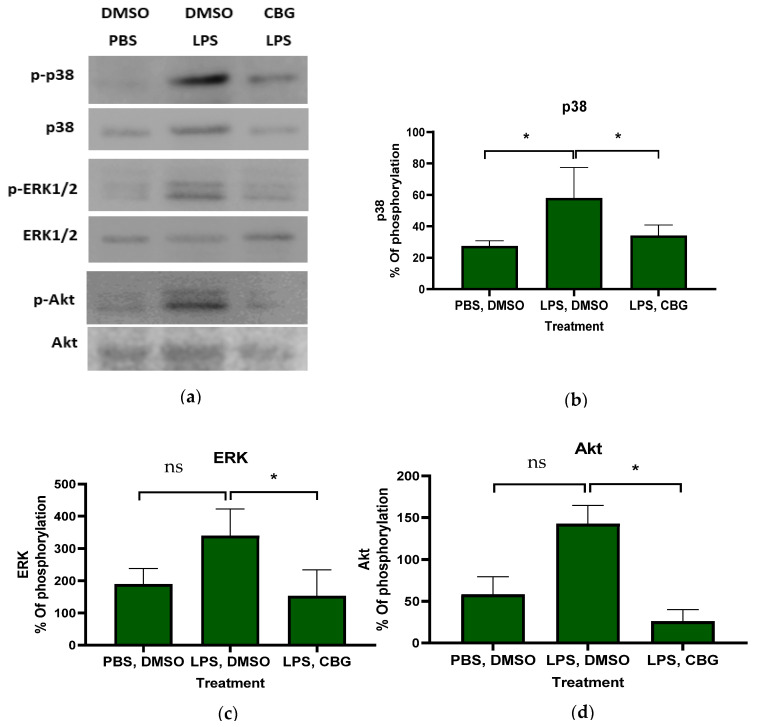
CBG attenuates inflammatory signaling in human neutrophils. Isolated CBG-treated neutrophils were LPS-activated for 60 min. Then, cells were harvested and lysed in 100 µL of 2× sample buffer containing β-mercaptoethanol. Phosphorylation levels of p38 MAPK, ERK1/2 and Akt were assessed by Western blot analysis using antibodies against phospho-p38 (Thr180/Tyr182), total p38, phospho-ERK1/2 (Thr202/Tyr204), total ERK1/2, phospho-Akt (Ser473) and total Akt (**a**). Densitometric quantification of phosphorylated proteins was normalized to the corresponding total protein levels for p38 (**b**), ERK1/2 (**c**) and Akt (**d**) as follows: *p*-protein/total corresponding protein) × 100%. Data are presented as the mean ± SD from three independent donors, expressed relative to LPS-activated neutrophils. Statistical analysis was performed using one-way ANOVA followed by Fisher’s LSD test; * *p* < 0.05 was considered statistically significant and ns was not.

To elucidate the mechanism underlying the inhibitory effect of CBG on neutrophils, primary human neutrophils were isolated, activated and treated with CBG. Phosphorylation levels of proteins within inflammatory signaling pathways were assessed and representative gels are shown (Figure 2a). CBG treatment resulted in attenuating p38 MAPK, ERK and Akt phosphorylation by 1.7- (34 ± 6.7, *p* = 0.05, *n* = 3, 95% CI (0.1, 47.9)), 2.2- (153 ± 83.1, *p* = 0.03, *n* = 3, 95% CI (20.78, 351.8)) and 4.5-fold (25 ± 14.2, *p* = 0.001, *n* = 3, 95% CI (72.4, 161.0) relative to the activated control treatment (LPS, DMSO), respectively (Figure 2b–d). Western blot gels for human neutrophils from three donors are provided (Supplementary Figure S3). These findings shed light on the underlying mechanism of CBG regulatory effects, demonstrating its ability to attenuate phosphorylation within the MAPK and Akt inflammatory signaling pathways. However, the small number of biological replicates limits generalizability.

### 2.3. CBG-Mediated Suppresion of IL-6 Is Enhanced by CB2 Receptor Modulation

**Figure 3:**
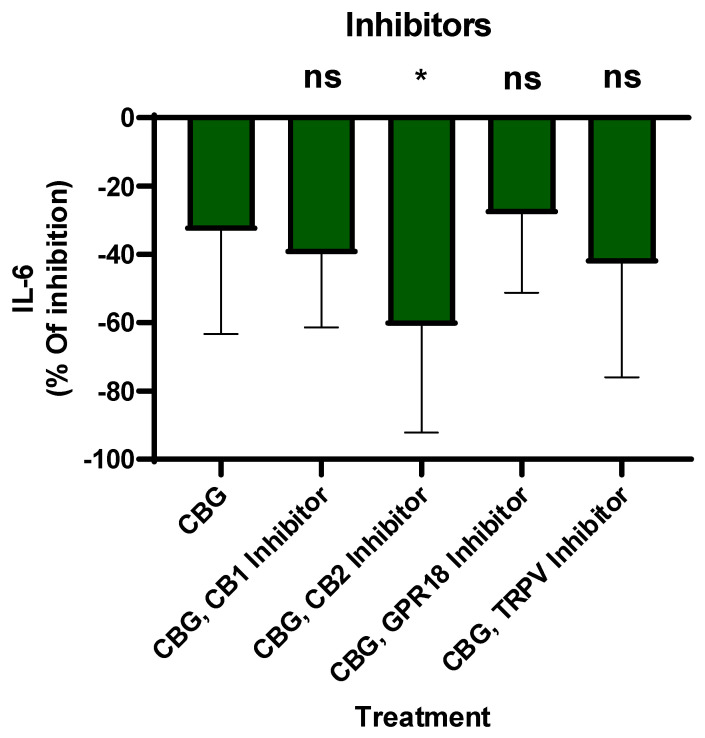
CBG suppresses IL-6, enhanced by CB2 receptor blockade. IL-6 secretion by LPS-activated neutrophils was considered 100%, and the percentage of inhibition was calculated relative to it. Data are presented as the mean ± SD from eight independent donors. Statistical analysis was performed using one-way ANOVA followed by Fisher’s LSD test; * *p* < 0.05 was considered statistically significant and ns was not.

CBG interacts with the endocannabinoid system and other key signaling pathways, such as CB1, CB2, TPR channels and GPR18, potentially influencing inflammation [26]. Critics often question whether the anti-inflammatory effects of cannabinoids stem from canonical receptor-mediated signaling or non-specific cellular activity. To address this, we conducted a series of functional inhibition assays using primary human neutrophils. By employing selective antagonists for the CB1, CB2 and GPR18 receptors and TRPV channels on neutrophils, inhibitors were introduced 30 min prior to CBG treatment. Then, LPS was added overnight and IL-6 secretion was tested by ELISA. The percentage of inhibition was calculated relative to LPS-activated neutrophils. The results indicate that CBG inhibits IL-6 levels by 33% ± 29.4 (Figure 3). However, the CBG and CB2 inhibitor together reduced IL-6 levels by 60% ± 29.4, *p* = 0.03, *n* = 8, 95% CI [2.8, 52.6]. Meanwhile, CBG and other receptor inhibitor reductions in IL-6 were not significant. These findings suggest that CBG-mediated suppression on IL-6 is enhanced in the presence of CB2 receptor inhibition, indicating a potential functional interaction between CBG activity and the CB2 receptor axis in regulating IL-6 secretion.

### 2.4. CBG Inhibits Neutrophil Migration Toward IL-8

Neutrophils are first-line responders to infections and are recruited to target tissues through the action of chemoattractant molecules, such as chemokines [51]. In humans, IL-8 (CXCL8) is a key chemokine for the chemotaxis of polymorphonuclear leukocytes, neutrophils and monocytes/macrophages when acting on CXCR1 and CXCR2 [52]. IL-8 exerts its pathogenetic action, promoting the detrimental activation of immune and stromal cells in the RA synovial membrane, tendons and extra-articular sites, causing extra-articular complications [53].

**Figure 4:**
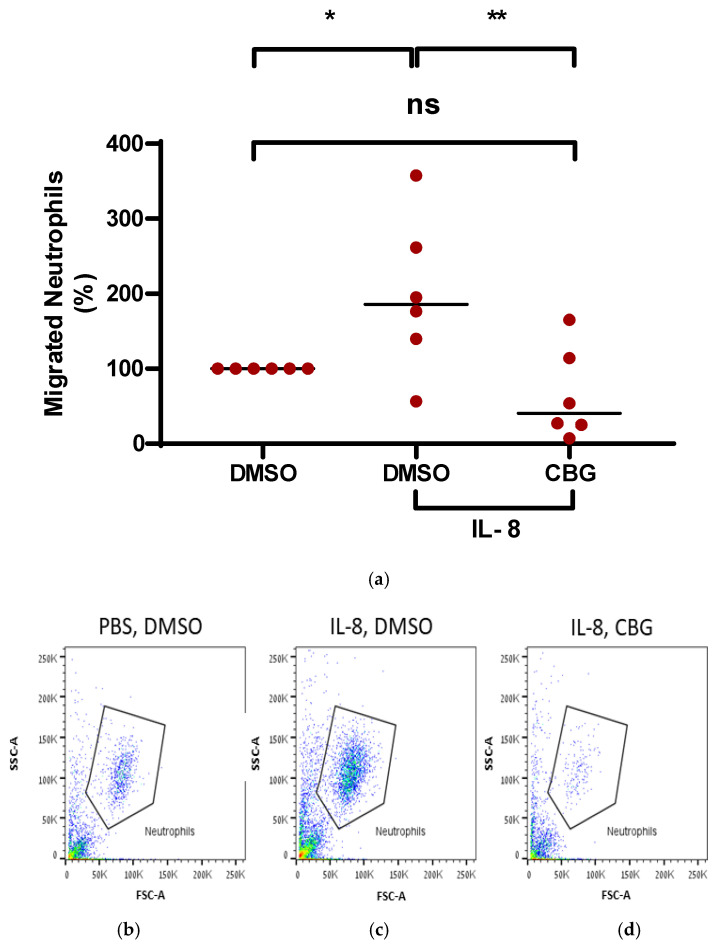
CBG inhibits neutrophil migration towards IL-8. CBG-treated human neutrophils were migrated to IL-8 through the Boyden chamber for 3 h. The percentage of migrated neutrophils was normalized to the control condition (**a**). Representative flow cytometry plots of neutrophil migration toward IL-8 following treatment with CBG (1 µg/mL) are shown (**b**–**d**). For migration quantification, cells in the lower chamber were harvested and analyzed via flow cytometry using a fixed time and a fixed volume. The time for the acquisition of each sample was fixed for 2 min and the volume of the FACS buffer was 200 µL in all samples. Also, isolated neutrophils were gated and identified by their characteristic FSC/SSC profiles, with doublets and debris excluded from the final count. Data represent means calculated from six healthy donors (black lines), with each dot representing an individual donor. Statistical analysis was performed by one-way ANOVA followed by Fisher’s LSD test, with * *p* < 0.05 and ** *p* < 0.01 considered statistically significant and ns considered not statistically significant.

Consequently, to examine whether CBG has the capacity to inhibit neutrophil migration toward IL-8, peripheral blood isolated neutrophils were isolated and treated with CBG. Their ability to migrate toward IL-8 across a Boyden chamber was examined. Neutrophils that migrated through the pores into the lower chamber were collected and subjected to flow cytometry analysis. IL-8 stimulated neutrophil recruitment by twofold compared to the control (197 ± 103, *p* = 0.02, *n* = 6, 95% CI [−183.3, −12.4]). Treatment with CBG significantly decreased the migration of neutrophils toward IL-8 back to baseline (65 ± 61.5, *p* = 0.004, 95 CI (47.01, 217.9), Figure 4a). Representative results for flow cytometry analysis are shown for three groups of treatment: PBS-DMSO, IL-8-DMSO and IL-8-CBG (Figure 4b–d).

Accordingly, these results support the notion that the anti-inflammatory effect of CBG is manifested by suppressing neutrophil recruitment to the site of inflammation and has the potential to mitigate RA severity.

### 2.5. CBG Attenuates Disease Severity in a Mouse Model of Rheumatoid Arthritis

Cannabinoids represent a novel class of anti-inflammatory agents effective against a range of inflammatory and autoimmune diseases driven by activated T cells and other immune components [54]. CBGA is the primary precursor for most of the cannabinoids, including CBD [26]. Previously published results demonstrate that a high-CBD extract modulates inflammation and immune cell activity in rheumatoid arthritis [25]. Consistent with these findings, the data presented in this study reveal that CBG directly regulates neutrophil inflammatory signaling, cytokine secretion and migratory capacity, cellular processes known to play a critical role in rheumatoid arthritis pathogenesis.

**Figure 5:**
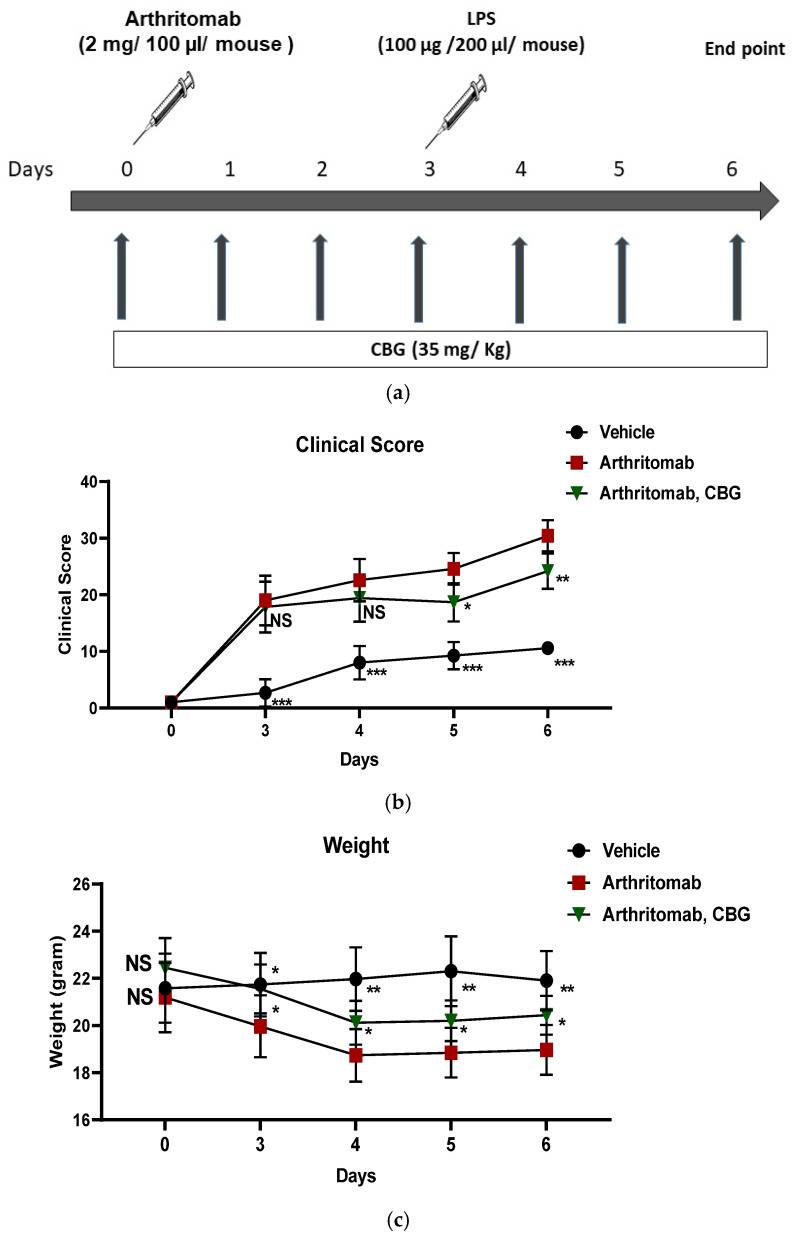
CBG reduces the clinical score and limits weight loss in a mouse model of rheumatoid arthritis. Rheumatoid arthritis was induced in C57BL/6 mice by the intraperitoneal injection of ArthritoMab (2 mg/100 µL per mouse; a cocktail of four monoclonal antibodies against type II collagen), followed by lipopolysaccharide (LPS; 100 µg/200 µL per mouse) administration on day 3. Arthritic mice were treated once daily with cannabigerol (CBG; 35 mg/kg) by sublingual administration (**a**). Clinical arthritis scores (**b**) and body weight (**c**) were monitored throughout the experiment. Mice were sacrificed on day 6. Data are the presented as mean ± SD for six mice per group (*n* = 6). Statistical analysis was performed using repeated measures two-way ANOVA followed by Fisher’s LSD test, with * *p* < 0.05, ** *p* < 0.01 and *** *p* < 0.001 considered statistically significant compared with the ArthritoMab + vehicle group. NS considered not statistically significant.

Together, these findings provided a strong rationale for investigating the therapeutic potential of CBG in a murine model of RA. Accordingly, C57BL/6 mice were induced with RA using ArthritoMab, a cocktail of four monoclonal antibodies directed against type II collagen. Rheumatoid arthritis-induced mice were treated with CBG once a day, sublingually (Figure 5a). The mice clinical score and body weight were monitored throughout the experiment. ArthritoMab-treated mice exhibited significantly higher clinical scores across the experimental period (29.5 ± 2.7, *p* < 0.001, *n* = 6, 95% CI (16.43, 23.23) day 6) compared with vehicle-treated controls. CBG treatment significantly reduced clinical scores in RA-induced mice on days 5 (18.6 ± 3.3, *p* = 0.015, *n* = 6, 95% CI (1.29, 10.5)) and 6 (24 ± 3.1, *p* = 0.008, *n* = 6, 95% CI (1.8, 10.6)) of the experiment (Figure 5b). ArthritoMab treatment also resulted in a pronounced reduction in body weight compared with control mice (19.5 ± 1.01, *p* = 0.0015, *n* = 6, day 6, 95% CI (−4.4, −1.4)), whereas CBG treatment significantly mitigated weight loss in RA-induced mice (20.4 ± 1.05, *p* = 0.02, *n* = 6, day 6, 95% CI (−2.6, −0.2), Figure 5c).

### 2.6. CBG Decreases Inflammatory Monocytes and Neutrophils in the Joints of a Murine Rheumatoid Arthritis Model

RA is developed through a series of events following a triggering event, which is the emergence of a chemokine for neutrophils in the synovium. Monocytes (macrophages) appear after neutrophil infiltration according to the natural course of inflammation and secrete IL-1β and TNFα, which stimulate synoviocytes, amplifying the inflammation [55].

**Figure 6:**
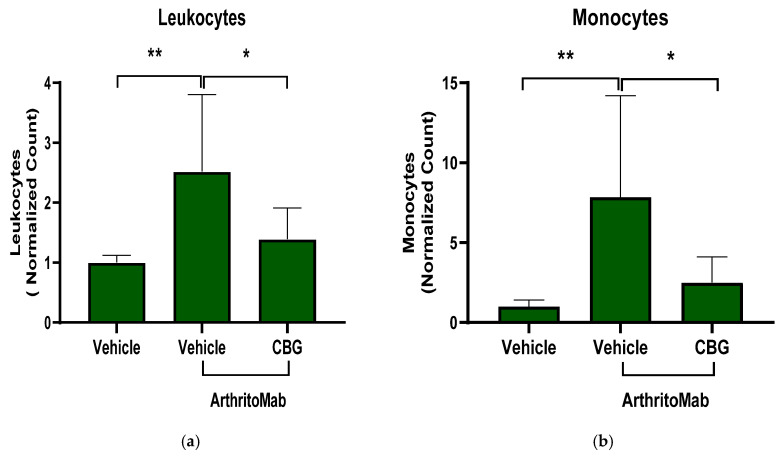

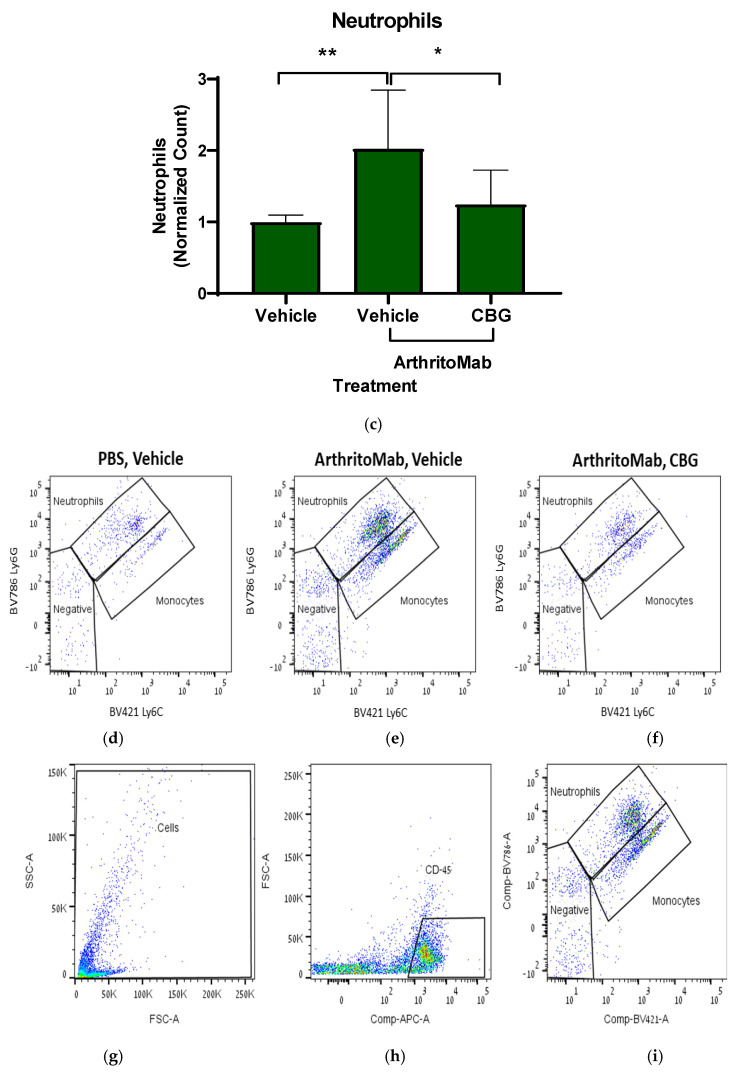
CBG decreases inflammatory monocytes and neutrophils in the joints of a murine rheumatoid arthritis model. Rheumatoid arthritis–induced mice were treated with CBG (35 mg/kg). On day 6, the mice were sacrificed and the joint tissues were harvested, homogenized, filtered and centrifuged to obtain single-cell suspensions. Cells were stained with anti-CD16/32 (Fc receptor blocker), APC–anti-CD45, BV421–anti-Ly6C and BV786–anti-Ly6G antibodies for flow cytometric analysis. Absolute counts of leukocytes, monocytes and neutrophils were quantified (**a**–**c**). Representative flow cytometry plots for the three treatment groups, PBS + vehicle, ArthritoMab + vehicle and ArthritoMab + CBG (**d**–**f**), and the flow cytometry gating strategy are shown (**g**–**i**). Data are presented as the mean ± SD for six mice per group (*n* = 6). Statistical analysis was performed using one-way ANOVA followed by Fisher’s LSD test, with * *p* < 0.05 and ** *p* < 0.01 considered statistically significant compared with the ArthritoMab + vehicle group.

In order to examine the effect of CBG on inflammatory cell recruitment to inflamed joints, the RA mice model was induced. RA-diseased mice were treated with CBG for six days, and then the mice were sacrificed and the joints were homogenized, filtered and centrifuged. Cells were stained with anti CD16/32 (FcR blocker), APC anti-CD45, BV421 anti-LY6C and BV786 anti-LY6G for flow cytometry analysis. The cell counts of leukocytes, monocytes and neutrophils were detected. ArthritoMab treatment increased leukocytes, monocytes and neutrophils levels in the joints by approximately 2.5-fold (2.4 ± 1.2, *p* = 0.005, *n* = 6, 95% CI (0.5, 2.5)), 8-fold (7.8 ± 6.3, *p* = 0.006, *n* = 6, 95% CI (2.2, 11.4)) and 2-fold (2 ± 0.8, *p* = 0.006, *n* = 6, 95% CI (0.3, 1.7)), respectively, compared with the controls (Figure 6a–c). However, CBG treatment reduced leukocyte (1.3 ± 0.5, *p* = 0.02, *n* = 6 95% CI (0.13, 2.1)), monocyte (2.3 ± 1.6, *p* = 0.02, *n* = 6, 95% CI (0.7, 9.9)), and neutrophil (1.2 ± 0.48, *p* = 0.02, *n* = 6, 95% CI (0.09, 1.4)) levels to near-baseline values in RA-diseased mice. Representative flow cytometry results for three groups, PBS-Vehicle, ArthritoMab-Vehicle and ArthritoMab- CBG (Figure 6d–f), and the flow cytometry gating strategy are shown (Figure 6g–i). The percentage of leukocytes, monocytes and neutrophils relative to the vehicle ArithritoMab group of CD45 leukocytes is shown for additional insight into immune composition changes (Supplementary Figure S4).

These findings demonstrate that CBG exerts a regulatory effect by limiting inflammatory immune cell recruitment to inflamed joints in RA-diseased mice, supporting its potential to downregulate pathogenic immune responses in rheumatoid arthritis.

### 2.7. CBG Attenuates Cytokine Secretion in a Rheumatoid Arthritis Model

**Figure 7:**
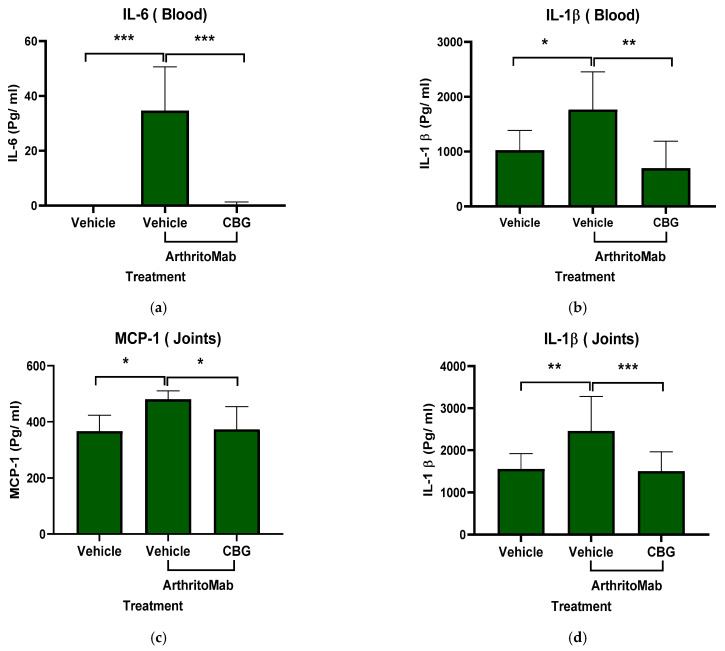
CBG hampers cytokine secretion in a rheumatoid arthritis model. Rheumatoid arthritis-induced mice were treated with CBG, and Cytokine levels of IL-6 and IL-1β were detected in the blood serum (**a**,**b**), as well as MCP-1 and IL-1β in joint homogenates (**c**,**d**), and quantified by ELISA. Data are presented as the mean ± SD for six mice per group (*n* = 6). Statistical analysis was performed using one-way ANOVA followed by Fisher’s LSD test, with * *p* < 0.05, ** *p* < 0.01 and *** *p* < 0.001 considered statistically significant compared with the ArthritoMab + vehicle group.

Inflammatory cytokines play a central role in the development and pathogenesis of rheumatoid arthritis, and biological therapies targeting these cytokines have revolutionized RA treatment [56]. To assess whether CBG can inhibit cytokine secretion, we used the ArthritoMab-induced mouse model of RA. Levels of inflammatory cytokines were measured in the blood and joints of RA-diseased mice. ArthritoMab treatment increased IL-6 and IL-1β levels in the blood by approximately 30-fold (34 ± 15.8, *p* < 0.001, *n* = 6, 95% (22.3, 47.03)) and 2-fold (1769 ± 683.6, *p* < 0.02, *n* = 6, 95% CI (86.21, 1401)), respectively (Figure 7a,b), and elevated MCP-1 and IL-1β levels in the joints by 1.25-fold (480 ± 30.2, *p* = 0.012, *n* = 6, 95% CI (29.5, 197.5)) and 1.6-fold (2460 ± 817.3, *p* = 0.002, *n* = 6, 95% CI (354, 1442)) compared with vehicle-treated controls (Figure 7c,d). Treatment with CBG significantly reduced the levels of these cytokines in both blood and joint tissues. These results indicate that CBG inhibits cytokine secretion in RA-diseased mice.

## 3. Discussion

Rheumatoid arthritis represents a severe condition that leads to the destruction of the affected joints and increasing disability. Rheumatoid arthritis (RA) is the prototype example of a chronic inflammatory joint disease, which shows a low probability of resolving spontaneously and usually requires the life-long control of inflammation by anti-rheumatic drugs [57].

The treatment goals for RA are to reduce the pain and stop/slow further damage. First-line treatment is based on non-steroidal anti-inflammatory drugs (NSAIDs) and corticosteroids that are used for pain and inflammation relief. Second-line treatment is slow-acting, disease-modifying anti-rheumatic drugs (DMARDs) like methotrexate and hydroxychloroquine, which are used to slow the disease’s progression. Newer medications available include more targeted therapies like biologics (e.g., rituximab) and Janus kinase (JAK) inhibitors (e.g., tofacitinib) [17].

However, Current conventional treatments sometimes fail or achieve only partial successes. In addition, RA drugs are associated with socioeconomic costs and undesirable side effects. Therefore, there is an unmet need for new and innovative therapeutic approaches. In this study, we demonstrated the immune-inhibitory capacity of CBG in modulating rheumatoid arthritis. Previous studies have shown the potential use of cannabinoids as a new class of anti-inflammatory agents against a number of inflammatory and autoimmune diseases like multiple sclerosis, colitis, liver injury, RA and cancer, which are primarily triggered by cellular immune components [54]. Moreover, studies have highlighted the therapeutic potential of non-psychoactive cannabinoids on coronavirus [58] and RA [23]. Furthermore, high-CBD extract has been reported to attenuate cytokine storm and enhance outcomes in acute lung disease [24] and asthma [59]. In addition, CBD has anti-inflammatory effects in a murine model of LPS-induced acute lung injury, and the augmentation of adenosine signaling through the adenosine A2A receptor is the most likely mechanism controlling the actions of cannabidiol [60]

Recent findings demonstrate that the high-CBD extract CBD-X shows anti-inflammatory effects in rheumatoid arthritis by reducing pro-inflammatory cytokine production, attenuating immune-cell migration, and inhibiting inflammatory signal transduction [25].

However, CBG is a precursor to most of the other cannabinoids; it is safe and well-tolerated substance that does not produce intoxicating or adverse effects, making it attractive to the clinic [61]. Unlike THC and CBD counterparts, CBG does not bind directly to the CB1 receptors in the brain, which are responsible for the psychoactive effects of cannabis [26]. Furthermore, CBG has an anti-inflammatory effect on the kidney tissue of rats subjected to a high-fat, high-Sucrose diet [29]. In addition, CBG demonstrated a regulatory effect on rheumatoid arthritis synovial fibroblasts [28].

While research into CBG is growing, it still lags behind that of other cannabinoids like CBD and THC. Our lab has extensive experience in immune-assays and cannabinoids; therefore, we were intrigued to expand the research into CBG’s regulatory effect on the immune system, especially on rheumatoid arthritis.

The pathogenic role of neutrophils in RA lies in the alteration of several processes, including increased cell survival and migratory capacity, abnormal inflammatory activity, elevated oxidative stress and an exacerbated release of neutrophil extracellular traps [19]. Neutrophils are believed to play an important role in both the initiation and progression of RA, and large numbers of activated neutrophils are found within both synovial fluid (SF) and synovial tissue from RA joints [62]. This makes Neutrophils key targets in rheumatoid arthritis (RA) treatment. Therefore, we initially examined the effect of CBG on human peripheral blood isolated neutrophils, focusing on its capacity to reduce the production of the key pro-inflammatory cytokines TNF-α and IL-6. Human neutrophils synthesize—at least in vitro—very low amounts of TNFα in response to the TLR agonist. However, they exert potent autocrine effects in amplifying neutrophil-derived cytokines and chemokines [63]. 

To assess whether CBG modulates inflammatory signaling, we examined its effects on LPS-stimulated human neutrophils. Our results show that CBG inhibits the phosphorylation of p38 MAPK, ERK and Akt, indicating the suppression of key inflammatory pathways [64,65]. However, a recent study showed that CBG treatment significantly diminished the expression of TLR-4 in collagen-induced RA in rats [66].

The suppression of key players like MAPK and PI3K/Akt, which are involved in NF-κB signaling, inhibit inflammation and cytokine secretion [50]. In addition, p38α stabilizes IL-6 [67] and TNF-α [68] mRNA via multiple AU-rich Elements. Consequently, CBG-mediated p38 inhibition leads to reduction in the stability of their AU-rich mRNA transcripts. Collectively, CBG inhibition of PI3K/Akt and MAPK leads to the downregulation of TNFα and IL-6. While reactive oxygen species (ROS) production [69], NET formation [70] or degranulation [71] assays were not tested, the signal transduction of Akt/MAPK axis inhibition by CBG disrupts the upstream signals that govern these processes.

The study by Lowin et al. (2023) found broad anti-inflammatory effects of CBG in RA synovial cells and PBMCs [28]. While both our studies show anti-inflammatory efficacy, we found that neutrophils are sensitive to lower concentrations of CBG than PBMCs. Furthermore, while we acknowledge the role of the TRPA1 up-stream receptor identified by Lowin [28], our research studied the ERK, P38 and Akt down-stream signaling and an engagement of the CB2 receptor axis. Figure 3 shows CBG inhibition and CB2 receptor inhibitor involvement in a selective way. Our results suggest that CBG has an inhibitory effect on neutrophil activation. By engaging the CB2 receptor axis, CBG initiates a signaling cascade that intercepts and downregulates the IL-6 pathway. This mechanism is situated downstream of TLR4, allowing CBG to effectively attenuate multiple inflammatory response upon activation. However, we have not seen CB2 inhibitor antagonism and we did not show loss-of-function models like CB2 receptor knockout mice. Therefore, CB2 receptor engagement is suggestive not definitive. Furthermore, since CBG has multiple binding targets, other receptors like PPARγ and α2-adrenoceptors that have not been tested yet can be suggested candidates.

The signaling blots have only *n* = 3 independent biological donors; while this is minimal, it is common for signaling assays to establish mechanistic proof-of-concept, as it accounts for inter-individual biological variability. A high degree of consistency was observed across all three donors, coupled with a sufficient statistical effect. Furthermore, these human findings are consistent with the anti-inflammatory outcomes observed in our in vivo CAIA model, providing cross-species validation of CBG’s efficacy. However, we acknowledge the small number of biological replicates that limits generalizability.

The cytokine CXCL8 (also known as IL-8) is known to be one of the most potent chemoattractant molecules that, among several other functions, is responsible for guiding neutrophils through the tissue matrix until they reach sites of injury [72]. IL-8 exert its pathogenetic action promoting the detrimental activation of immune and stromal cells in RA synovial membrane, tendons, and extra-articular sites, as well as blood vessels and lungs, causing extra-articular complications, which might be excluded by the action of anti-TNFα and anti-IL6R targeted therapies [53]. Therefore, we assessed CBG’s effect on neutrophil migration induced by IL-8. CBG significantly inhibited neutrophil recruitment toward IL-8.

While the inhibition of neutrophil migration could stem from a reduction in surface CXCR1/2 expression [73], our data suggest a negative regulation of the downstream signaling. Neutrophil recruitment to the arthritic joint is a multi-step process requiring precise cytoskeletal remodeling and chemotaxis, both of which are governed by the Akt [74] and MAPK signaling axes [75]. By attenuating these pathways, CBG significantly reduces the recruitment of neutrophils from the microvasculature into the synovial space. This reduction in local neutrophil density subsequently diminishes the release of cartilage-degrading proteases and pro-inflammatory cytokines, thereby attenuating the progression of joint destruction. Consequently, the observed inhibition of IL-8-mediated neutrophil migration in vitro suggests an explanation for the reduced synovial infiltration seen later in our in vivo CAIA model.

The cytotoxic and pro-inflammatory properties of neutrophils are likely mediators for RA inflammation and tissue destruction [14]. These results prompt us to evaluate the therapeutic potential of CBG in mitigating rheumatoid arthritis in mice model. CBG improved the clinical score and weight for RA-induced mice. In addition, CBG has an inhibitory effect on leukocytes like monocytes and neutrophils in RA-induced joints. Studies show that a low dose of CBG demonstrates antioxidant and anti-inflammatory capacity ending in the reduction in cytokine secretion, thereby decreasing the inflammatory state [76].

CBG was administered sublingually at a dose of 35 mg/kg. This route was selected for the special anatomical and physiological features of the oral mucosa—specifically, its extensive vascularization and direct connection to systemic circulation. Therefore, this rout might contribute to CBG avoiding first-pass metabolism within the gastrointestinal tract and liver [77]. Furthermore, this route provides a non-invasive, translationally relevant method of administration without inducing the stress response associated with repeated injections.

While the CAIA model is an appropriate tool for evaluating antibody-driven inflammation and neutrophil recruitment, we acknowledge that it primarily represents the effector phase of RA. Unlike the Collagen-Induced Arthritis (CIA) model, CAIA bypasses the initial loss of self-tolerance and the subsequent T and B cell-mediated priming phases [78]. The 6-day experimental duration was specifically selected to capture the peak effector phase of the CAIA model. This window allowed for a precise evaluation of CBG’s ability to intercept cytokine secretion and cell recruitment. However, we acknowledge that this short-term model does not capture the chronic stages of RA.

The reduction in synovial neutrophil infiltration observed in CBG-treated mice likely results from a combination of impaired recruitment and attenuated chemoattractant gradients, rather than enhanced apoptosis. Our in vitro data demonstrate that CBG inhibits the migration of neutrophils toward IL-8. Furthermore, the simultaneous reduction in cytokines in both the blood and the joints in the CAIA model suggests that CBG lowers the overall cytokine secretion, thereby weakening the systemic-to-local cytokine gradient that drives continuous neutrophil influx. Importantly, cell viability remained high in our primary cultures, suggesting that CBG modulates neutrophil behavior without inducing premature programmed cell death.

While the observed reductions in joint tissue MCP-1 and IL-1β (1.25- and 1.6-fold) may appear modest in magnitude, they have a significant biological effect. IL-1β is a key player and pro-inflammatory cytokine that has been implicated in pain, inflammation and autoimmune conditions [79]. Marginal reductions in master regulators like IL-1β can lead to a disproportionate attenuation of downstream inflammatory cascades. Given that MCP-1 is a primary driver of monocyte and neutrophil chemoattraction [80], a 20–25% decrease in its local concentration may be sufficient to push the recruitment signal below the threshold required for sustained leukocytic infiltration. Accordingly, the modulation of cytokine levels by CBG in the blood and the joints leads to observable clinical improvements in RA-diseased mice.

Regarding the pharmacokinetics of CBG, it was not the focus of our study. However, a recent study shows the in vivo metabolism and pharmacokinetics of CBG in horses after IV and oral administration. The findings highlight extensive metabolite formation with significant glucuronidation, a large distribution volume and high clearance [81].

Furthermore, regarding the safety of CBG at a dose of 35 mg/kg in mice, recent toxicological and pharmacokinetic research used almost similar doses of CBG 30 mg/kg by intravenous (i.v) administration, CBG produced little to no intoxicating tetrad effects (catalepsy or hypothermia) and was well-tolerated in C57BL/6Crl mice [82]. From a translational perspective, the 35 mg/kg mouse dose used in our study scales to approximately 2.8 mg/kg in humans (based on BSA allometric scaling). A double-blind, placebo-controlled, cross-over field trial indicates that 20 mg of hemp-derived CBG reduces subjective ratings of anxiety and stress in healthy cannabis-using adults in the absence of motor or cognitive impairment, intoxication or other subjective drug effects (e.g., heart palpitations, dry mouth) [83].

Previous clinical studies have demonstrated that cannabis-based medicines can significantly improve pain quality and reduce disease activity in RA patients [84]. Synovial pathology reports reveal lymphocyte-predominant infiltration in most RA cases, with synovial neutrophils (SNs) observed in only 30% of patients. This finding suggests that neutrophil involvement in RA pathogenesis is not universal but subtype-specific, potentially linked to distinct clinical phenotypes [38]. Therefore, the inhibitory effect of CBG on neutrophils shows its therapeutic potential. While our findings provide an appropriate rationale for the therapeutic use of CBG in neutrophil-mediated RA, the translational implications must be framed with caution. Human RA is a highly heterogeneous and chronic condition involving the interaction of various innate and adaptive immune cells, which secrete various chemokines, inflammatory mediators and other substances that act on the patient’s synovial tissue and joints [85]. Therefore, while CBG represents a promising lead for attenuating acute joint inflammation, further long-term clinical studies are necessary to determine its efficacy across the various clinical phenotypes and chronic stages of human disease.

Importantly, we acknowledge the limitations of this study; First, while the use of primary human-isolated neutrophils ensures high translational relevance, the small number of donors (*n* = 3) in the Western blot assay may not fully capture the genetic and clinical heterogeneity in humans. However, the high degree of consistency in the Akt/MAPK signaling suppression across these independent donors suggests a significant biological effect. Second, while the CAIA model is ideal for studying the acute inflammatory process, it does not account for the chronic autoimmune priming involving T and B cell memory. Future studies using the Collagen-Induced Arthritis (CIA) model are needed to assess CBG’s impact on long-term disease modification. Third, while we did not perform de novo pharmacokinetic analysis, our sublingual dosing of 35 mg/kg was informed by recent literature demonstrating that such doses achieve systemic concentrations capable of modulating inflammatory targets without inducing toxicity [82]. Fourth, our results demonstrate that CBG immune regulation is consistent with the CB2 receptor signaling axis. However, more research should be conducted since CBG acts across several targets, including PPARγ and α2-adrenoceptors, that have not been tested yet. Fifth, given the multi-target nature of CBG, potential off-target effects cannot be ruled out. Finally, although we utilized a neutrophil-driven CAIA model and we disrupted neutrophil inflammatory functionalities by CBG, more experiments should be conducted to determine the neutrophil depletion effect on RA-diseased mice to establish them as primary targets.

**Figure 8:**
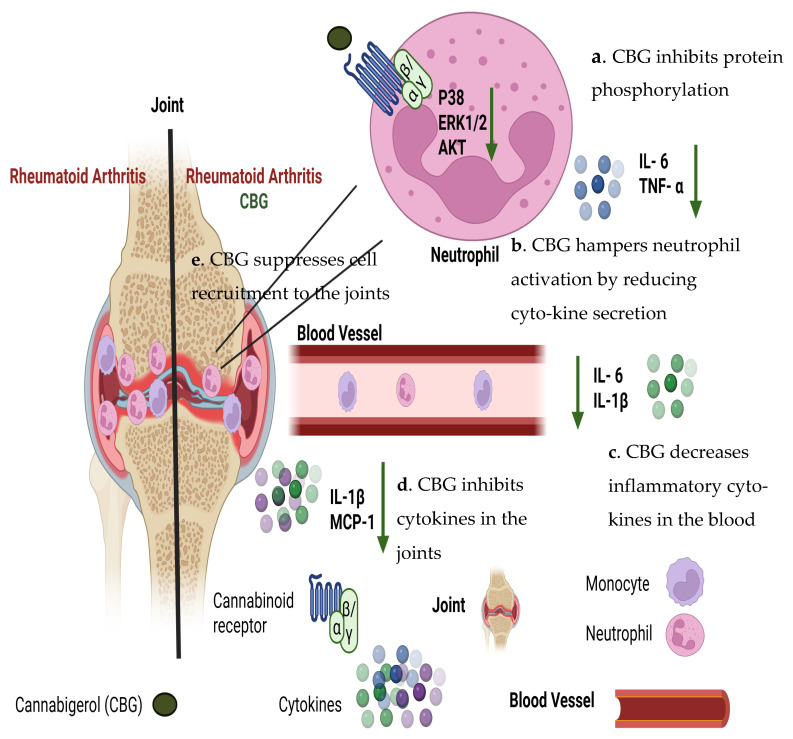
Proposed effects of CBG in an inflamed joint of an RA model. CBG inhibits P38, ERK1/2 and Akt phosphorylation (**a**). CBG hampers neutrophil activation by reducing the secretion levels of IL-6 and TNF-α (**b**). In addition, CBG decreases pro-inflammatory cytokines IL6 and IL-1 β in the blood (**c**). Furthermore, CBG inhibits IL-1 β and MCP-1 cytokines in the joints (**d**). Consequently, CBG suppresses the recruitment of immune cells like neutrophils and monocytes to the joints (**e**). Collectively, these findings suggest an anti- inflammatory profile for CBG and indicate its potential therapeutic relevance in RA. Illustration created at 24 March 2026 by BioRender. Rambam, C. (2026) (https://BioRender.com/d8kjsu4).

To sum this up, our study demonstrates that CBG exerts multi-level regulatory effects on neutrophils, key contributors to rheumatoid arthritis pathogenesis (Figure 8).

## 4. Materials and Methods

### 4.1. Reagents

ELISA kits for human IL-6 and TNF-α and ELISA kits for mouse Il-6, IL- 1β and MCP-1 were purchased from BioLegend (San Diego, CA, USA). TruStain FcX™ (rat anti-mouse CD16/32, IgG2a, λ), APC anti-mouse CD45 (rat, IgG2b, κ, clone 30/F11), Brilliant Violet 785 anti-mouse Ly-6G (Rat IgG2a, κ, clone 1A8) and Brilliant Violet 421 anti-mouse Ly-6C (Rat IgG2c, κ) were purchased from BioLegend (CA, USA). ArthritoMab was purchased from MD Bioproducts (Zurich, Switzerland). The medium X-VIVO 15 with gentamicin and phenol red for neutrophil cells was obtained from Lonza (Basel, Switzerland). The Human Neutrophil Isolation kit was obtained from STEMCELL Technologies (Vancouver, BC, Canada). Lipopolysaccharide (LPS) was obtained from Santa Cruz (Santa Cruz, CA, USA). Fetal bovine serum (FBS), glutamine, penicillin and 100 U/mL streptomycin were obtained from Biological Idustries (Beit Haemek, Israel).

### 4.2. Cannabis

The Cannabigerol (CBG) used in this study was provided by Raphael Pharmaceutical, Inc. (Las Vegas, NV, USA). The compound was verified to be 98% pure via High-Performance Liquid Chromatography (HPLC). For the ex vivo experiments, a primary stock solution of CBG was prepared at a concentration of 1 mg/mL by dissolving the crystalline solid in DMSO. To prevent degradation and maintain conformational stability, the stock was protected from light and stored at −20 °C. Experimental dosing concentrations of 1 and 2 µg/mL were prepared by serial dilution of the stock into the medium. To ensure the observed therapeutic potential was solely due to CBG, a vehicle control containing the equivalent concentration of 0.1–0.2% DMSO was utilized in all assays. This specific dosage range was selected based on the viability assays (Supplementary Figure S2). For the in vivo experiments, CBG was first dissolved in absolute ethanol and subsequently blended with Kolliphor in a 1:7 ratio (12.5% ethanol and 87.5% Kolliphor). A vehicle-only control was administered to the control group to isolate the therapeutic effects of the CBG molecule. Mice were administered a dose of 35 mg/Kg. This dosage was delivered in a controlled volume (15 µL) placed under the tongue using a micro-pipette to ensure rapid systemic entry.

### 4.3. Human Peripheral Blood Samples

Human peripheral blood samples were obtained from the Israeli National Biobank for Research (MIDGAM) at Rambam Health Care Campus. The experiments were authorized by the Helsinki Committee at Rambam Health Care Campus (Authorization No. 0442–20 RMB). Venous blood was obtained from healthy donors from both genders at age range of 21–70.

### 4.4. Mice

C57BL/6 mice were obtained at the age of 8 to 10 weeks from Envigo, Israel. All mice were housed at a barrier/free and specific pathogen/free animal facility at the Pre-Clinical Research Authority, Technion-Israel Institute of Technology in Haifa, Israel. All experiments were performed according to the regulations of the Inspection Committee on the Constitution of the Animal Experimentation of the Technion-Israel Institute of Technology in Haifa, Israel, from which authorization for performing animal studies was provided (Authorization No. IL-0840521). Experiments conformed to the regulations in the Prevention of Cruelty to Animals Law (Experiments on Animals) 5754–1994 and the Prevention of Cruelty to Animals Rules (Experiments on Animals) 5761–2001, correct as of 1 December 2005. Prior to the induction of arthritis, mice were assigned to experimental groups (control, RA, RA-CBG) using a simple randomization protocol generated by a lottery. To ensure an unbiased outcome assessment, a double-blind protocol was implemented. experiment solutions were prepared and coded by Dr. Antonina Pechkovsky, a researcher not involved in the experimental analysis. Furthermore, Dr. Miran Aswad performed the daily clinical assessments, and the data analysis was blinded to the treatment assignments. The clinical score assessment included appearance, body function, behavior, vocalization and arthritis paw score assessments.

### 4.5. Isolation of Immune Cells from Blood

Peripheral blood samples of healthy volunteers were collected in EDTA-containing tubes. Neutrophils were isolated from blood samples by negative magnetic selection with the EasySep Direct Human Neutrophil Isolation Kit (STEMCELL Technologies) according to the manufacturer’s instructions (purity~95%, (Supplementary Figure S1)). Briefly, Isolation Cocktail (50 µL/mL) and RapidSpheres (50 µL/mL) were added to a whole blood sample tube for 5 min. The sample tube was topped up with the recommended medium and inserted into the magnet for 10 min. Then, the sample was transferred to a new tube and RapidSpheres (50 µL/mL) were added for an additional 5 min incubation. The sample with the RapidSpheres was inserted into the magnet for 5 min and the sample was transferred to a third tube. The last step was repeated to obtain a final clear fraction.

### 4.6. Cell Culture and Treatment of the Cells with CBG

In total, 200,000 isolated human neutrophil cells were treated with 1–2 µg/mL CBG or DMSO as a control for two hours. After incubation, CBG-treated cells were centrifuged and activated with 100 ng/mL LPS overnight. The cells were centrifuged, supernatants were collected and levels of TNF-α and IL-6 levels were detected from neutrophils by ELISA with a BioTek ELISA plate reader (Shoreline, WA, USA). The experiment has been conducted in four technical replicates—thirteen biological replicates for TNF-α and fifteen biological replicates for IL-6.

### 4.7. Cell Viability Measurement

CBG-treated neutrophils were washed, and the medium was added to the cells with 10% Alamar Blue solution. As a negative control, Alamar Blue was added to the medium without cells. The cells were further incubated for another six hours at 37 °C. The absorbance of the test and control wells was read at 570 nm and 600 nm with a BioTek ELISA plate reader.

### 4.8. Western Blot Analysis for Human Primary Isolated Neutrophils

Neutrophils were isolated from healthy donors by negative magnetic selection with the EasySep Direct Human neutrophil Isolation Kit (Vancouver, BC, Canada). Isolated neutrophils were activated with 100 ng/mL LPS and treated with 1 µg/mL CBG or DMSO as a control. After 45 min, the cells were collected and pelleted by centrifugation at 18,800× *g* and washed twice with ice-cold PBS. The washed cell pellets were resuspended in 100 µL 2× Laemmli Sample Buffer with added β-mercaptoethanol at a ratio of 1:20 (SB + βME), incubated at 100 °C for 5 min and centrifuged at 18,800× *g* for 5 min; the cell pellet containing debris was discarded. The protein samples were separated on a 10% SDS-PAGE gel and transferred onto nitrocellulose membrane using a Trans-Blot Turbo Transfer Apparatus (Bio-Rad, Hercules, CA, USA), following the manufacturer’s instructions. The membrane was blocked with 5% BSA in Tris-buffered saline containing 0.1% Tween 20 followed by incubation overnight at 4 °C with total or phospho-specific antibodies to P38 (1:1000), ERK1/2 (1:1000) and Akt (1:1000) in 5% BSA in Tris-buffered saline containing 0.1% Tween 20. The blots were washed with Tris-buffered saline containing 0.1% Tween 20 and incubated with the respective secondary antibodies conjugated with horse radish peroxidase for one hour at room temperature. The blots were washed with Tris-buffered saline containing 0.1% Tween 20. Immunoreactive proteins were detected with the Enhanced Chemiluminescence (ECL) kit (Thermo scientific, Waltham, MA, USA). The relative density of the protein bands was scanned using Image Quant LAS4000 (GE HealthCare, Chicago, IL, USA) and analyzed by Image-J 1.8.0–172. The experiment has been conducted in three biological replicates [86].

### 4.9. Cell Treatment with CBG and Cannabinoid Receptors

Isolated human neutrophils were incubated with cannabinoid inhibitors including CB1 (JD-5037, 0.5 µM), CB2 (BML-190, 100 µM), GPR18 (PSB-CB5, 1 µM) and TRPV (Ruthenium red, 10 µM) for 30 min. The cells were washed and CBG (1 µg/mL) was added and incubated for 2 h. Human neutrophils were centrifuged and the medium was replaced with X-VIVO 15 media with L-glutamine and gentamicin. For stimulating proinflammatory conditions, LPS (100 ng/mL) was added and cultured at 37 °C and 5% CO_2_ overnight. The cells were centrifuged, supernatants were collected and IL-6 levels were detected by ELISA. The experiment has been conducted in three technical replicates and eight biological replicates.

### 4.10. Neutrophil Migration

For the cell migration experiments, 3 µm 24-well Boyden chambers were used. A total of 250,000–500,000 cells/mL of neutrophils were seeded in the upper chamber of the 24-well Boyden chamber in the medium. In the lower chamber, 100 ng/mL of hIL-8 were placed. CBG (1 μg/mL) or DMSO as a vehicle were added to the cells. Three hours later, cells that migrated through the pores into the lower chamber were collected and counted via flow cytometry. The experiment has been conducted in two biological replicates and six technical replicates.

### 4.11. Collagen Antibody-Induced Arthritis Mouse Model

A cocktail of four monoclonal antibodies to type II collagen (ArthritoMab; 2 mg/100 µL/mouse) was injected intravenously at day 0. Mice in the PBS group were injected with equal volumes of sterile PBS.

At day three, all animals except the PBS group were injected with LPS (100 μg/200 µL/mouse), intraperitoneally. The vehicle (ethanol and Kolliphor in ratio 1:7) or CBG (35 mg/Kg) were administered once a day, sublingually. The clinical score and mice weight were monitored during the experiment days. At day six, the mice were euthanized by 5% Isoflurane and CO_2_ and blood was drained from the heart collected for ELISA. Alternatively, joints were collected and disassembled by the gentleMACS™ Dissociator (3 legs, 2 mL PBS, program B then C, Miltenyi Biotec, Bergisch Gladbach, Germany). Fluids were filtered twice through a mesh paper or strainer during centrifugation. Supernatants were centrifuged twice and collected for ELISA. Cells were stained with anti CD16/32 (FcR blocker), APC anti- CD45, BV786 anti-LY6G and BV421 anti-LY6C for flow cytometry analysis using High Throughput LSRFortessa (BD Biosciences, San Jose, CA, USA). The experiment has been conducted in two technical replicates; each group has three mice, and in total, there are six biological replicates for each treatment group.

### 4.12. ELISA

ELISA kits have been used according to manufacturer’s instructions. One day prior to running the ELISA, Capture Antibody was diluted in 1X Coating Buffer. In total, 100 μL of this Capture Antibody solution was added to all wells of a 96-well plate. The plates were sealed and incubated overnight (16–18 h) between 2 °C and 8 °C. The plates were washed four times with at least 300 μL Wash Buffer (PBST) per well and blot residual buffer by firmly tapping the plate upside down on absorbent paper. To block non-specific binding and reduce the background, 200 μL 1X Assay Diluent was added per well. The plates were incubated at RT for 1 h. The plates were washed four times with Wash Buffer. In total, 100 μL/well of standards or samples were added to the appropriate wells. The plates were incubated at RT for 2 h. The plates were washed four times with Wash Buffer. 11. In total, 100 μL of diluted Detection Antibody solution were added to each well, and the plates were incubated at RT for 1 h. In total, 100 μL of diluted Avidin-HRP solution were added to each well, and the plates were incubated at RT for 30 min. The plates were washed five times with Wash Buffer. In total, 100 μL of TMB Substrate were added and incubated in the dark for 10–15 min. Positive wells turned blue in color. The reaction was stopped by adding 100 μL of Stop Solution to each well. Positive wells should turn from blue to yellow. Absorbance was read at 450 nm and 570 nm within 15 min. The absorbance at 570 nm was subtracted from the absorbance at 450 nm [87].

### 4.13. Flow Cytometry

For joint samples, fluorochrome-conjugated antibodies (mentioned above) were incubated in the dark for 20 min. The cells were washed, resuspended in 2% FBS in PBS, and 2% paraformaldehyde-PBS fixation buffer was added for flow cytometry analysis by a BD LSRFortessa system (BD Biosciences, San Jose, CA, USA) [88].

### 4.14. Statistical Analysis

Statistical data presentation and experimental design: All in vitro and in vivo data were presented as the mean ± standard deviation, *p* value, sample size (*n*) and 95% confidence intervals (CIs); the ex vivo experiments data were analyzed in comparison to the activated treatment as a baseline. The significance in neutrophils was determined in comparison to the DMSO-LPS group. The data were analyzed by one- way ANOVA and for neutrophils (Fisher’s LSD test with values *p* < 0.05 considered statistically significant (* *p* < 0.05, ** *p* < 0.01, *** *p* < 0.001)).

In the in vivo acute experiments, data for the CAIA model were analyzed in comparison to Arthitomab-induced mice as a baseline named the “Arthitomab, Vehicle” group. Each experiment included six mice (*n* = 6) in each group. The data were analyzed by one-way ANOVA (Fisher’s LSD test with values *p* < 0.05 considered statistically significant (* *p* < 0.05, ** *p* < 0.01, *** *p* < 0.001)).

### 4.15. Illustration

This illustration was created using https://www.biorender.com/.

## 5. Conclusions

Our study demonstrates that CBG exerts a regulatory effect on neutrophils, key contributors to rheumatoid arthritis pathogenesis (Figure 8). CBG inhibits the secretion of pro-inflammatory cytokines TNF-α and IL-6 from human peripheral blood isolated neutrophils and suppresses the activation of key inflammatory signaling pathways, including ERK, p38 MAPK and Akt. Additionally, CBG impairs neutrophil chemotaxis toward IL-8, limiting their recruitment to inflamed tissues. In vivo, CBG treatment in a murine RA model reduced inflammatory monocytes, and neutrophil infiltration into the joints lowered circulating and local cytokine levels and ameliorated disease severity, as reflected by improved clinical scores and body weight.

There are some future directions we look forward to applying in order to strengthen this study—for instance, identifying specific receptors responsible for CBG inhibition and, furthermore, testing CBG in a chronic model and examining it with a combination therapy like DMARDs.

So far, these findings highlight CBG as an effective preclinical modulator candidate for affecting neutrophil-mediated immune responses and attenuating inflammation in rheumatoid arthritis.

## Data Availability

The original contributions presented in this study are included in the article/Supplementary Materials. Further inquiries can be directed to the corresponding author.

## Supplementary Materials

The following supporting information can be downloaded at: https://www.mdpi.com/article/10.3390/ph19040560/s1, Supplementary Figure S1. Isolated human blood neutrophils purity by flow cytometry. Supplementary Figure S2. Effect of CBG on human neutrophil viability. Supplementary Figure S3. Western blot gels for human neutrophils from three donors. Supplementary Figure S4. Percentage of leukocytes, monocytes and neutrophils in the joints of CBG-treated rheumatoid arthritic mice.

## Abbreviations

The following abbreviations are used in this manuscript:

RA	Rheumatoid arthritis
CBG	Cannabigerol
CAIA	Disease-modifying anti-rheumatic drugs
DMARDs	Disease-modifying anti-rheumatic drugs
NSAIDs	Non-steroidal anti-inflammatory drugs
NETs	Neutrophil Extracellular Traps
JAK	Janus kinase
