# Supplementary material for: Cannabigerol (CBG) Modulates Neutrophil Activity and Ameliorates Rheumatoid Arthritis Pathogenesis
Source: Pharmaceuticals (Basel). 2026 Mar 31;19(4):560. doi: 10.3390/ph19040560 (PMC13118493; doi:10.3390/ph19040560)
Supplement: Supplementary file 1 [file pharmaceuticals-19-00560-s001.zip › pharmaceuticals-4164215-supplementary.pdf]

Article

Supplementary Material:

Cannabigerol (CBG) Modulates Neutrophil Activity and Ameliorates Rheumatoid Arthritis Pathogenesis

Miran Aswad 1, Antonina Pechkovsky 1, Haya Hamza 1 and Igal Louria-Hayon 1,2,3,*

1. The Shanti Center for Medical Cannabis Research, Rambam Health Care Campus, Haifa 3109601, Israel
2. Clinical Research Institute at Rambam (CRIR), Rambam Health Care Campus, Haifa 3109601, Israel
3. The Division of Research at Rambam, Rambam Health Care Campus, Haifa 3109601, Israel

* Correspondence: i_louriahayon@rambam.health.gov.il

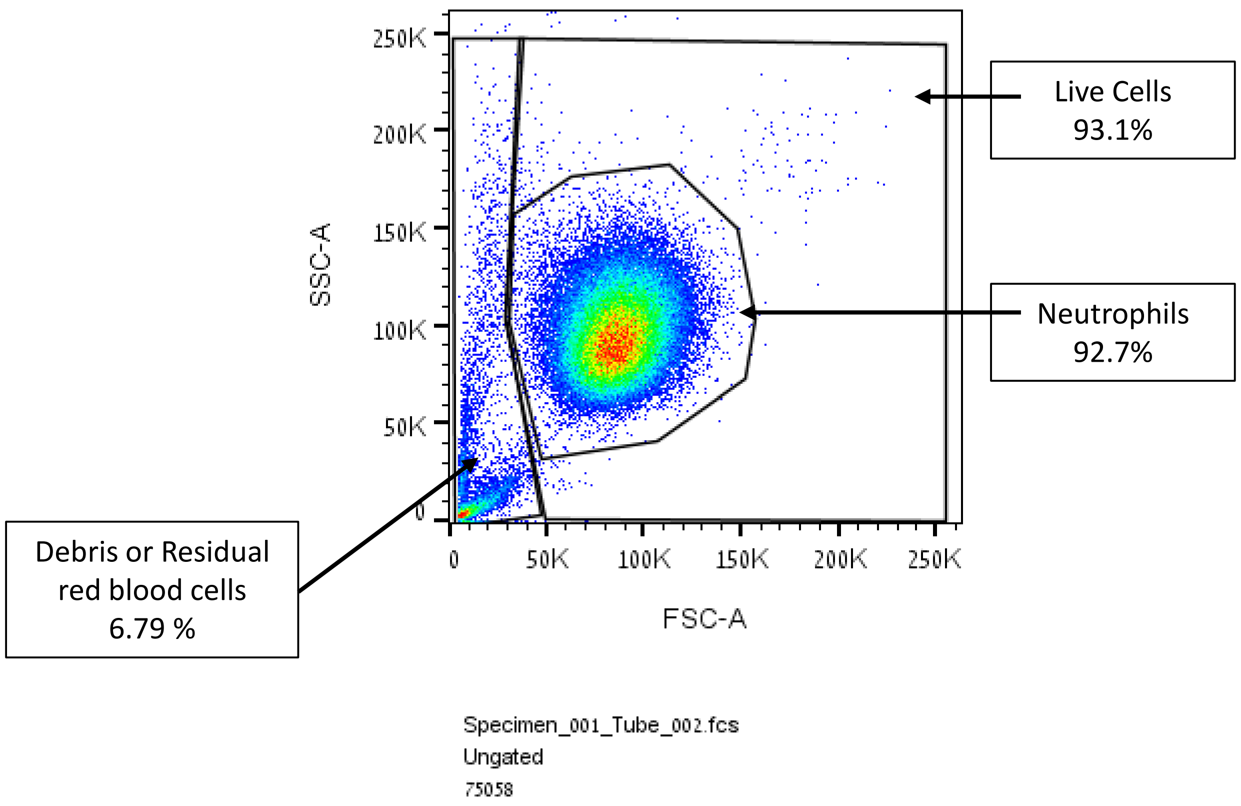

Supplementary figure S1. Isolated human blood neutrophils purity by flow cytometry. Human blood neutrophils were purified with EasySep direct human neutrophil isolation Kit and analyzed with FSC-A vs SSC-A gate by flow cytometry. Representative percentage of isolated cells are shown.

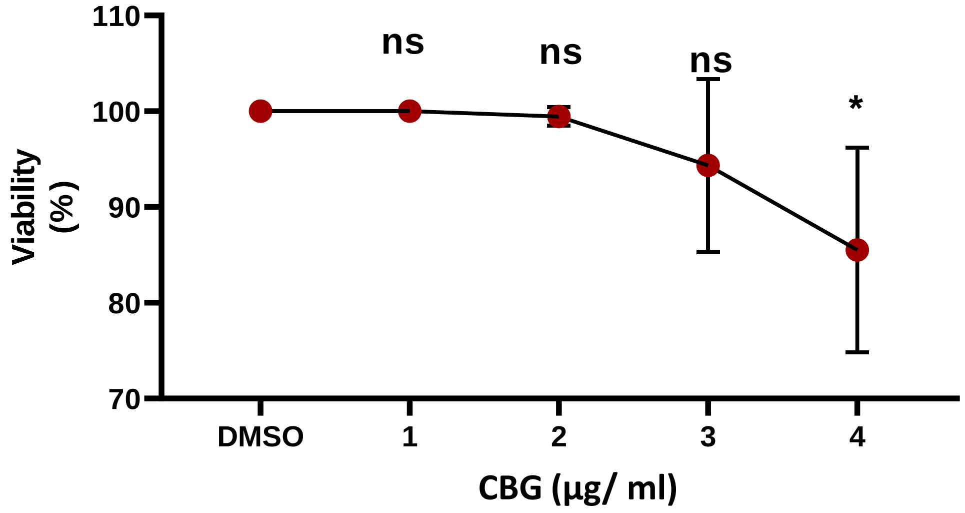

Supplementary figure S2. Effect of CBG on human neutrophil viability. Isolated human neutrophils were treated with increasing concentrations of cannabigerol (CBG; 1, 2, 3, and 4 µg/mL) or vehicle (DMSO) for 2 h in serum-free X-VIVO™ 15 medium. Cells were then centrifuged, and fresh medium containing 10% Alamar Blue solution was added. Alamar Blue added to medium without cells served as a negative control. Cells were further incubated for 6 h at 37 °C, after which absorbance was measured at 560 nm and 590 nm using a spectrophotometer.

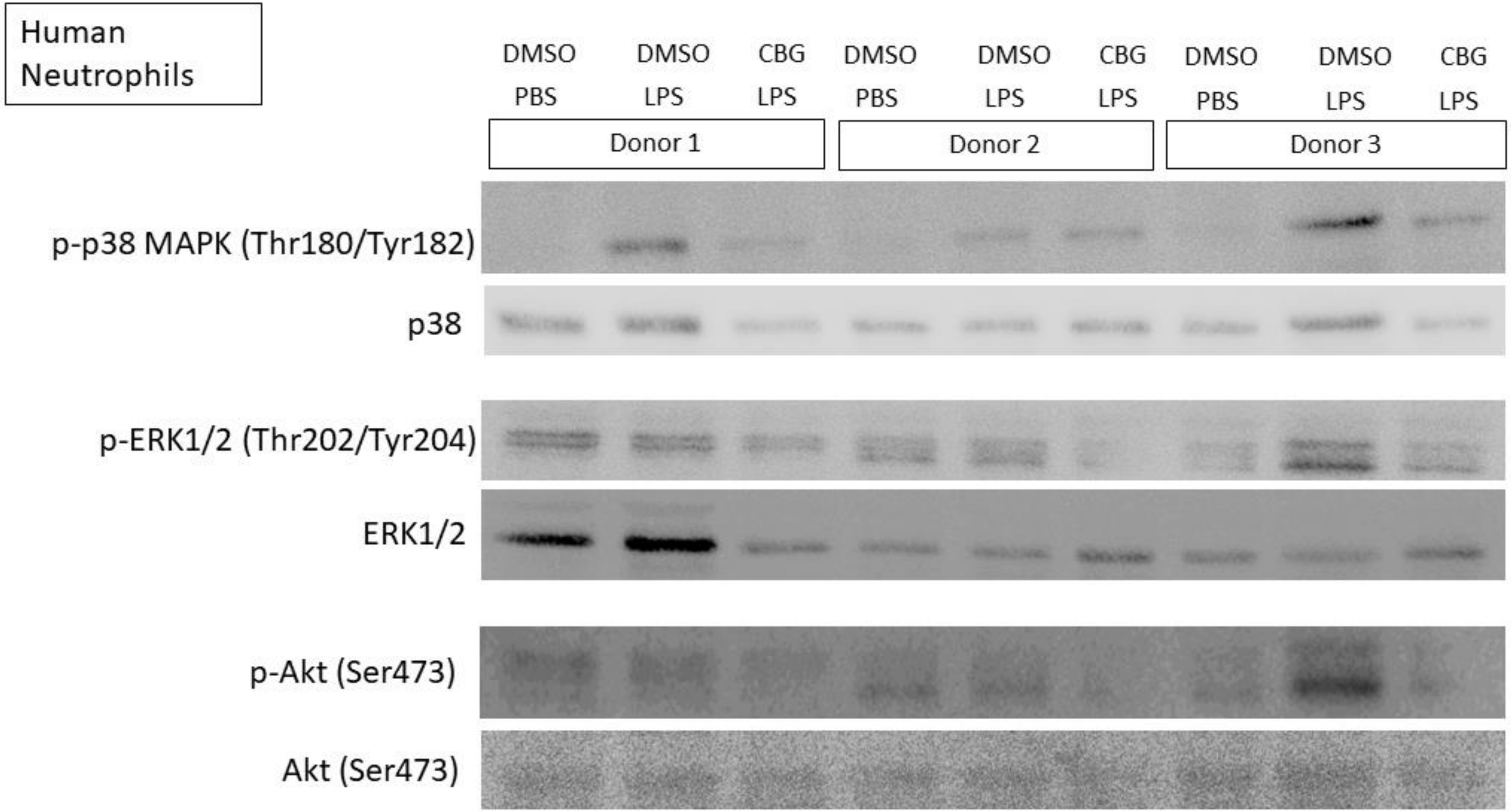

Supplementary Figure S3. Western blot gels for human neutrophils from three donors. Primary human neutrophils were isolated and activated with lipopolysaccharide (LPS; 1 µg/mL) and subsequently treated with cannabigerol (CBG; 1 µg/mL) or vehicle (DMSO) as a control. After 60 min, cells were harvested and lysed in 100 µL of 2× sample buffer containing β-mercaptoethanol. Phosphorylation levels of p38 MAPK, ERK1/2, and Akt were assessed by Western blot analysis using antibodies against phospho-p38 (Thr180/Tyr182), total p38, phospho-ERK1/2 (Thr202/Tyr204), total ERK1/2, phospho-Akt (Ser473), and total Akt. Original images are provided in a zipped file named "western blot gels".

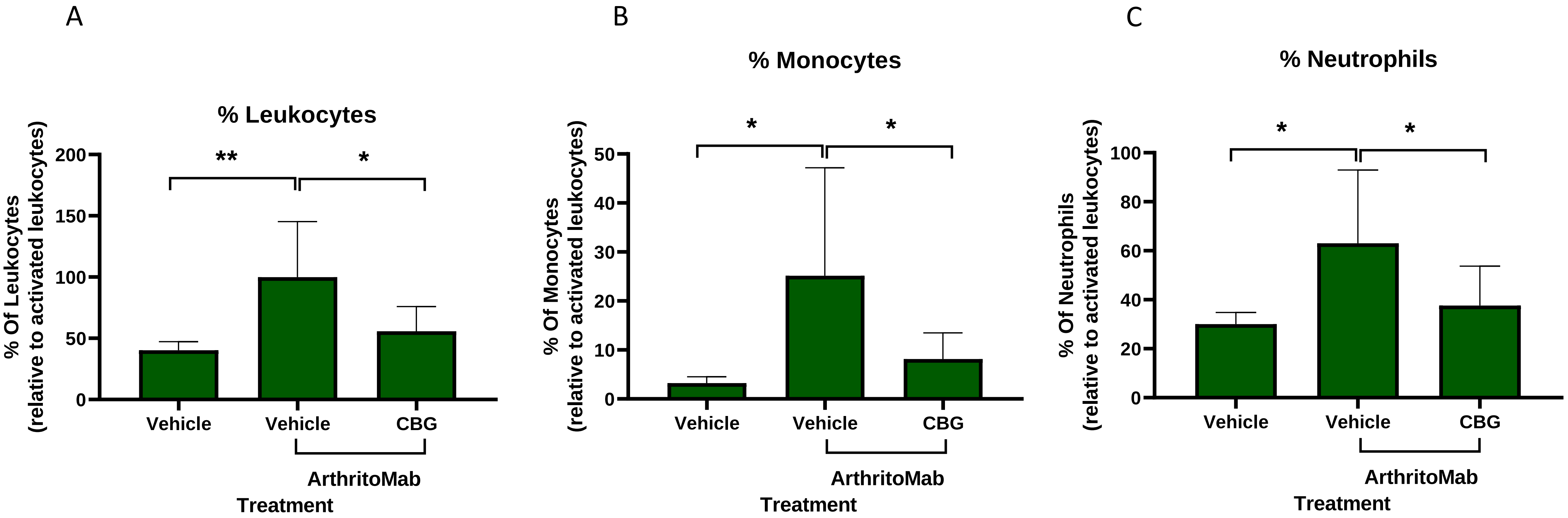

Supplementary Figure S4. Percentage of leukocytes, monocytes and neutrophils in the joints of a CBG- treated rheumatoid arthritic mice. Rheumatoid arthritis–induced mice were treated with CBG (35 mg/kg). On day 6, mice were sacrificed and joint tissues were harvested, homogenized, filtered, and centrifuged to obtain single-cell suspensions. Cells were stained with anti-CD16/32 (Fc receptor blocker), APC–anti-CD45, BV421–anti-Ly6C, and BV786–anti-Ly6G antibodies for flow cytometric analysis. Percentage of leukocytes, monocytes, and neutrophils were quantified relative to vehicle, ArithritoMab group of CD45- leukocytes (A–C). Data are presented as mean ± SD for six mice per group (n = 6). Statistical analysis was performed using one-way ANOVA followed by Fisher's LSD test, with $*p \leq 0.05$, $**p \leq 0.01$ and $***p \leq 0.001$ considered statistically significant.
